# Gamma Irradiated *Rhodiola sachalinensis* Extract Ameliorates Testosterone-Induced Benign Prostatic Hyperplasia by Downregulating 5-Alpha Reductase and Restoring Testosterone in Rats

**DOI:** 10.3390/molecules24213981

**Published:** 2019-11-04

**Authors:** Qi Xin, Mi-Jin Kwon, Ju-Woon Lee, Kwan-Soo Kim, Hao Chen, Maria G. Campos, Rosa Tundis, Cheng-Bi Cui, Young Ho Cho, Hui Cao

**Affiliations:** 1 Key Laboratory of Changbai Mountain Biological Resources and Functional Molecular Education, Yanbian University, Yanji 133002, China; qixin812@sina.com; 2 Division of Efficiency Evaluation of Biomolecules, PSA Co., Ltd., Daejeon 35365, Korea; kwonmj1108@naver.com (M.-J.K.); sjwlee@naver.com (J.-W.L.); 3 Greenpia Technology Inc., Yeoju-si 12619, Gyeonggi-do, Korea; kwanskim9@nate.com; 4 Suite 18B Sea View Plaza, 18 Tai Zi Road Shekou, Shenzhen 518067, China; haochen@siae.cn; 5 Observatory of Herb-Drug Interactions, Faculty of Pharmacy, University of Coimbra, Heath Sciences Campus, Azinhaga de Santa Comba, 3000-370 Coimbra, Portugal; mgcampos@ff.uc.pt; 6 Coimbra Chemistry Centre (CQC, FCT Unit 313) (FCTUC), University of Coimbra, Rua Larga, 3000-370 Coimbra, Portugal; 7 Department of Pharmacy, Health and Nutritional Sciences, University of Calabria, Via P. Bucci, 87036 Rende, Italy; rosa.tundis@unical.it; 8 Department of Pharmaceutics and Biotechnology, Konyang University, Daejeon 35365, Korea; 9 Guangdong-Macau Traditional Chinese Medicine Technology Industrial Park Development Co., Ltd., Hengqin New Area, Zhuhai 519031, China; 10 School of Pharmacy, Chengdu University of Traditional Chinese Medicine, Chengdu 611137, China

**Keywords:** *Rhodiola sachalinensis*, testosterone, benign prostatic hyperplasia, 5-alpha reductase

## Abstract

The effect of *Rhodiola sachalinensis* Boriss extract irradiated with 50 kGy gamma rays (HKC) on benign prostatic hyperplasia (BPH) was investigated. Seven-week-old male SD rats received a subcutaneous injection of 20 mg/kg of testosterone propionate (TP) to induce BPH. Then, the testosterone only group received testosterone, the testosterone + finasteride group received testosterone and finasteride (5 mg/kg), the testosterone + HKC group received testosterone and HKC extract (500 mg/kg). Prostate weight and the dihydrotestosterone (DHT) levels in serum or prostate tissue were determined. The mRNA expressions of 5-alpha reductase (AR) in prostate tissue were also measured. Compared to the control group, prostate weight was significantly improved in the TP group and decreased in the HKC and finasteride-treated groups. Furthermore, the mRNA expression of 5-AR in the prostate was significantly reduced in the HKC and finasteride-treated groups. Similarly, the expression levels of α-smooth muscle actin (α-SMA) and cytokeratin, which are associated with prostatic enlargement in the HKC and finasteride groups, were much lower than in the TP group. HKC treatment showed similar efficacy to finasteride treatment on rats with testosterone-induced BPH. HKC may be explored as a potential new drug for BPH treatment.

## 1. Introduction

Benign prostatic hyperplasia (BPH) is characterized by the nonmalignant overgrowth of the prostatic tissue and stromal cells within the transitional zone and periurethral area [1,2]. Although testosterone levels decreased with age could benefit this pathology [3], the major factor is 5-alpha reductase (5-AR), which improves the dihydrotestosterone (DHT) level [4].

BPH patients need to be treated via surgery or medication [5,6]. 5-AR inhibitors and α-adrenergic receptor blockers are mail medications. The natural sources are available to drug discovery for various complications of BPH [7,8]. For example, the tomato and black raspberry extracts help prevent and treat BPH by regulating the expression of DHT and 5-AR [9,10].

However, even if a material is found to improve prostate enlargement from natural products, it takes a long time to clarify the mechanism [11,12]. Therefore, considering that prostate hyperplasia is a long-standing disease according to aging, it is best to find a material that can prevent and treat enlargement of the prostate by effectively taking it safely for a long period.

In recent years, irradiation with high energy levels has been evaluated as an important technology for biorecycling research through structural transformation of food and biological materials, removal of harmful substances, and improvement of functionality. Therefore, the development of natural food and pharmaceutical materials using convergence technology of radiation and biotechnology is rapidly applying to industrialization. In other words, it is widely used in food processing, as well as long-term storage of food materials, through irradiation of appropriate dose, playing a big role in facilitating the industrial application of natural products.

*R. sachalinensis* has received great interest in the phytochemical investigation for many years, and many bioactive components have been isolated from it, such as phenylpropanoids, flavonoids, tannins, and so on [13]. *R. sachalinensis* has shown various bioactivities, including anticancer activity, antioxidant activity, anti-inflammatory activity, cardioprotective effect, and neuroprotective effects [14]. Moreover, salidroside, rosavins, and *p*-tyrosol in this plant possess benefits on fatigue, depression, and cognitive dysfunction [15,16].

Salidroside has shown many bioactivities, including antioxidant activity, antiaging activity, anticancer activity, anti-inflammatory activity, resisting anoxia, and cardioprotective effects. Recently, salidroside has been known as a cyclooxygenase-2 (COX-2) inhibitor, and some studies on the anti-inflammatory mechanism have been also reported [17,18]. Nonetheless, no studies have been described on the efficacy and therapeutic effects of these compounds on prostatic hyperplasia. In addition, we found that *R. sachalinensis* extract, as well as the bioactive components, such as salidroside, rosavin, rosarin, and *p*-tyrosol, had no proliferation inhibitory effect on prostatic hypertrophy cells (data not shown). Therefore, this study aimed to investigate the effects of the ethanol extract of *R. sachalinensis* irradiated with 50 kGy gamma rays (HKC) on prostatic hyperplasia using a testosterone propionate (TP)-induced BPH rat model.

## 2. Results

### 2.1. Effects on Activity of Aspartate Transaminase (AST), Alanine Aminotransferase (ALT), and Blood Urea Nitrogen (BUN)

**Figure 1:**
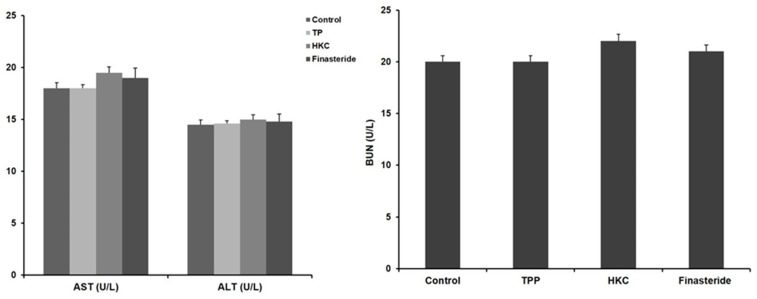
Effects of HKC (*R. sachalinensis* Boriss extract irradiated with 50 kGy gamma rays) on the activity of aspartate transaminase (AST)/alanine aminotransferase (ALT) and blood urea nitrogen (BUN). Data were expressed as the mean ± SE (*n* = 8).

HKC was safe as there were no significant differences in alanine aminotransferase (ALT) and aspartate transaminase (AST) activities among all of the groups. Similarly, blood urea nitrogen (BUN) was not significantly different from the control group, so it did not affect renal toxicity in HKC intake (Figure 1).

### 2.2. Effects of HKC on Prostate Weight (PW) and PW Index in TP-Induced BPH Rats

**Figure 2:**
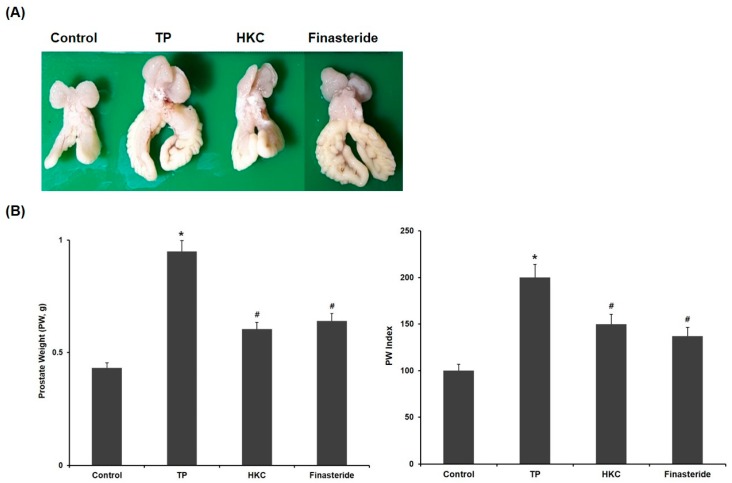
HKC extracts restore testosterone propionate (TP)-induced prostate enlargement. (**A**) Effect of HKC extract on prostate weight in rats with TP-induced benign prostatic hyperplasia (BPH), (**B**) Prostate weight and prostate index is the ratio of prostate weight to body weight. Date represented as mean ± SE (*n* = 8). Significant differences at * *p* < 0.01 compared with the control group. Significant differences at # *p* < 0.05 compared with the TP group.

The changes in prostate tissues in the control group and experimental group are shown in Figure 2. Relative prostate weight was used to evaluate the development of BPH. The rats treated with TP showed a significant increase in prostate weight (PW, 0.95 ± 0.02 g) and prostate weight/body weight ratio (PW index) compared to the control group (0.43 ± 0.09 g). In comparison with the TP-only treated group, HKC treatment groups (0.61 ± 0.08 g) significantly decreased the prostate weight gain induced with TP and decreased the PW index. The positive control group treated with finasteride also showed significant changes in prostate weight (0.64 ± 0.11 g) and PW index both. There were no significant changes in body weight.

### 2.3. Histopathological Examination

**Figure 3:**
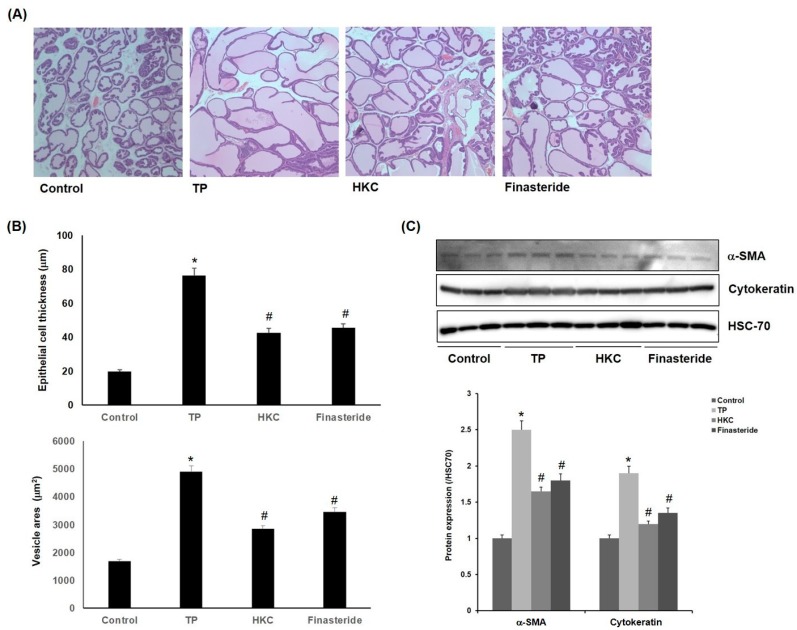
HKC extracts inhibit TP-induced prostate histopathological changes. (**A**) TP-induced rats’ prostatic tissues were stained with H & E staining for histological examination (magnification, 100×). Representative photomicrographs of prostate sections are shown. (**B**) Epithelial cell thickness and vesicle areas were calculated. Date represented as mean ± SE (*n* = 8). Significant differences at * *p* < 0.01 compared with the control group. Significant differences at # *p* < 0.05 compared with the TP group. (**C**) Protein expression of α-smooth muscle actin (α-SMA) and cytokeratin was measured using western blot analysis.

The control group showed the normal histological architecture of the prostate. The TP-treated group showed epithelial hyperplasia with a prostatic acini area as well as enlarged blood vessels. Co-treatment with HKC attenuated the pathological alterations induced by TP. HKC-treated group (42.5 ± 3.7 µm, 2840 ± 212 µm^2^) and finasteride-treated group (45.2 ± 2.8 µm, 3450 ± 153 µm^2^) also showed reductions in epithelial thickness (Figure 3A,B). It suggested that the HKC used in this study was capable of controlling prostate weight and the thickness of prostate tissue. One of the causes of enlargement of the prostate is due to the unequal hyperplasia of prostate stromal cells, which can be confirmed by the increased expression of α-smooth muscle actin (α-SMA) or cytokeratin in activated stromal cells. As shown in Figure 3C, the expression of α-SMA and cytokeratin was increased by TP treatment in the prostatic hypertrophy-induced group, but it was decreased by administration of the extract of HKC and finasteride.

### 2.4. Effects of 5-AR mRNA Expression

**Figure 4:**
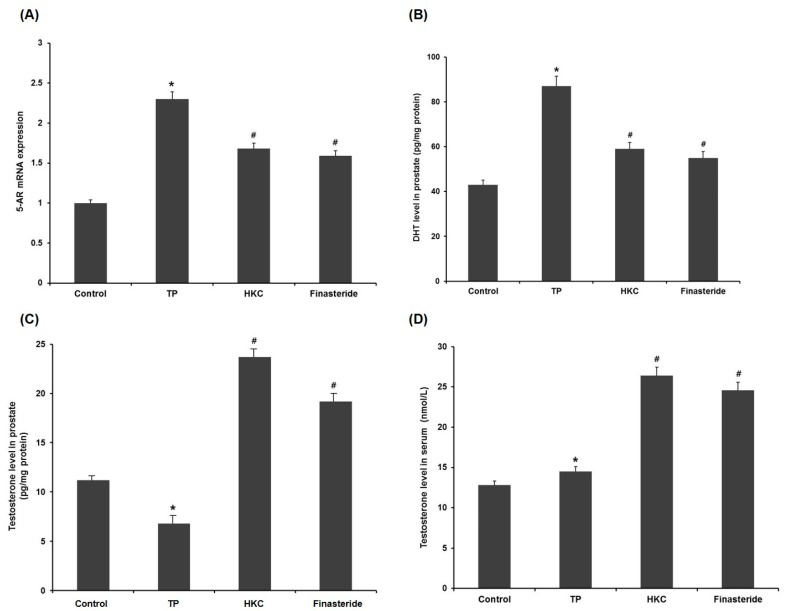
HKC extracts inhibit the conversion of testosterone to DHT via reducing 5-alpha reductase (5-AR) mRNA expression. (**A**) The effect of HKC extracts on 5-AR mRNA expression levels was detected by real-time RT-PCR. The effect of HKC extracts on the levels of testosterone (**C**, **D**) and DHT (**B**) in serum and prostatic tissue was detected by ELISA analysis. Date represented as mean ± SE (*n* = 8). Significant differences at * *p* < 0.01 compared with the control group. Significant differences at # *p* < 0.05 compared with the TP group.

In the pathogenesis of BPH, testosterone was transferred to DHT by the activation of 5-AR in the prostate. TP administration significantly increased mRNA of 5-AR compared to the control group. Co-administration of HKC reduced this TP-mediated elevation from the TP-treated group. The finasteride-treated group also showed a down-regulated expression of 5-AR (Figure 4A).

### 2.5. Effect on DHT and Testosterone Levels in Serum and Prostate

HKC was found to inhibit 5-AR activity by measuring testosterone levels in serum and prostate tissue (Figure 4C,D). Compared to the BHP group, the administration of finasteride improved the serum and prostate testosterone levels (Figure 4B). HKC restored the testosterone levels in serum and prostate in a similar manner as by finasteride, signifying that the HKC may act through inhibition of 5-AR.

## 3. Discussion

Beyond simple sterilization means gamma irradiation is a useful technology for a functional improvement through structural changes or physiological properties of natural ingredients, such as new production of derivatives of molecules [19]. Herein, the inhibitory effects of HKC on the development of BPH in rats were investigated. Rats with TP-induced BPH showed an increased relative prostate weight, elevated DHT levels and prostatic epithelial hyperplasia, and overexpressed 5-AR mRNA. However, we found that oral administration of HKC in the study, according to the dosage, was effective in preventing the progression of BPH caused by testosterone. Treatment with HKC inhibited the development of BPH, which was observed by the lowered relative prostate weights, inhibited expression of 5-AR mRNA, and reduced DHT levels in serum and prostatic tissue. HKC influenced prostatic epithelial hyperplasia from histological results. In this study, BPH induced by DHT markedly increased in the prostate volume, which is consistent with previous studies [20,21]. In contrast, as the concentration of testosterone in the prostate tissue increased, it was also reported that the hyperplasia of the prostate was induced [22,23].

HKC treatment significantly decreased the relative prostate weight. Histological examination of prostate tissues paralleled the results of prostate weight measurement. In support of these results, histological findings indicated that the administration of HKC remarkably attenuated the prostatic epithelial hyperplasia, which reduces prostate size. These results are consistent with finasteride used as a conventional and current treatment for the pathology under investigation in this work. We found that HKC administration reduced TP-mediated 5-AR mRNA expression, such as the finasteride-treated group showed a down-regulated expression of 5-AR. These results showed that testosterone blockade of DHT conversion increased testosterone levels compared to the TP-treated group. Though the mechanism of 5-AR is complex, inhibition of 5-AR by current BPH medications, such as finasteride, we used as positive control results in decreased conversion of testosterone to DHT. As 5-AR is the initial trigger of prostatic hyperplasia [23], our results suggested a pharmaceutical potential of HKC extracts on BPH treatment. Further investigation will be carried on to optimize the dose-activity for better control of efficacy and safety, as recommended in the WHO guidelines, for the standardized crude extracts, or even the isolated compounds.

## 4. Materials and Methods

### 4.1. Extracts Preparation

The dried roots of *R. sachalinensis* were provided by Professor Cui Chengbi of Yanbian University. After grinding, 100 g powdered roots were extracted twice with 70% (*v*/*v*) ethanol (1 L) at 70 °C for 24 h. The two parts were combined, filtered, and concentrated using a rotary evaporator (Eyela, Koishikawa Bunkyo, Tokyo, Japan) to obtain the aqueous extract of *R. sachalinensis*. Then, it was freeze-dried and kept at 4 °C for the following experiments.

### 4.2. Gamma Irradiation

The powder obtained from the dry extract of *R. sachalinensis* roots was dissolved in water at a concentration of 100 mg/mL. The solution was irradiated in a Co-60 irradiator (Nordion International, Ottawa, ON, Canada). The source strength was ca. 100 kCi with a dose rate of 5 kGy h-1 at 12 ± 0.5 °C. The dosimetry was performed using 5 mm diameter alanine dosimeters (Bruker Instruments, Billerica, MA, USA), and the free radical signal was measured using a Bruker EMS 104 EPR analyzer (Bruker Instruments, Billerica, MA, USA). After irradiation, it was freeze-dried and kept at 4 °C for the following experiments.

### 4.3. Experimental Procedures

The animals were randomly divided into the required experimental groups. To induce BPH, all experimental groups, except normal control group, were subcutaneously injected with 20 mg/kg body weight of testosterone propionate (TP) dissolved in the sterilized corn oil for 4 weeks. After 1 week of acclimatization, the rats were randomly divided into four groups (*n* = 7 per group): (A) Normal control group (NC group), corn oil injection (subcutaneously, s.c.); (B) TP group (BPH group), TP (20 mg/kg)/corn oil injection (s.c.); (C) experimental group (HKC-treated group), TP (20 mg/kg)/corn oil injection (s.c.) + HKC administration (500 mg/kg,); (D) positive control group (finasteride-treated group), TP (20 mg/kg)/corn oil injection (s.c.) + finasteride administration (10 mg/kg). All materials were administered to rats once daily for 4 weeks, and body weight was measured weekly. After the final treatment, all rats were fasted overnight and were anesthetized with a respiratory anesthetic. Blood samples were drawn from the caudal vena cava, and serum was stored at −80 °C for biochemical assays. The prostates were removed immediately and weighed. Relative prostate weight was calculated as the ratio of prostate weight to body weight, and this was expressed as a prostate index (PI).

The length of the experimental period was 4 weeks. During the experiment, animals were freely fed with a general diet and were provided with distilled water ad libitum. Each rat was raised in a separate stainless cage that was kept at a temperature of 24 ± 1 °C with a relative humidity of 50 ± 10% and an alternating 12 h day and 12 h night environment. Bodyweight was measured twice a week at a designated time. All experiments met the guidelines of the animal-testing ethics committee of Chonbuk National University (Approval Code: CBNU 2017-0116).

### 4.4. Measurement of Blood Biochemical Parameters

The serum samples were obtained by centrifugation at 2500× *g* for 10 min (4 °C) for the measurement of blood biochemical parameters. AST, ALT, and BUN levels were measured by detection kits (Roche, Basel, Switzerland).

### 4.5. Preparation of Prostate Homogenates

Prostatic tissue was homogenized (1/10 *w*/*v*) in tissue lysis/extraction reagent (Sigma-Aldrich, St. Louis, MO, USA) containing protease inhibitor cocktail (Roche, Mannheim, Germany) using a homogenizer. Homogenates were centrifuged at 15,500× *g* for 20 min at 4 °C. Total protein concentrations in the supernatant fractions were measured using BCA protein reagent (Thermo Fisher Scientific, Waltham, MA, USA).

### 4.6. ELISA for the Measurement of DHT and Testosterone Levels

The levels of testosterone and DHT in the serum or prostatic tissue were determined using an ELISA kit according to the manufacturer’s instructions. The absorbance was measured at 450 nm using a microplate ELISA reader (BioTek Instruments, Inc., Winooski, VT, USA). Values were expressed per mL for serum and per mg protein for the prostatic tissue.

### 4.7. Histological Examination

Fixed prostatic tissue embedded in paraffin wax was cut into 4 μm thick sections and stained with hematoxylin and eosin. Coverslips were mounted on sections using a mounting medium (Invitrogen, Carlsbad, CA, USA), and then the sections were examined under a microscope (Nikon, Tokyo, Japan).

### 4.8. Western Blot Analysis

Equal amounts of total prostatic protein (30 µg) were heated at 100 °C for 5 min, loaded onto 10–12% SDS-PAGE and electrophoresed. The proteins were then transferred to an Immobilon-P polyvinylidene difluoride membrane (Millipore Corporation, Burlington, MA, USA) using Transfer System (Bio-Rad Laboratories, Hercules, CA, USA). After blocking nonspecific binding sites with 5% nonfat milk at room temperature for 1 h, membranes were incubated with the primary antibodies overnight at 4 °C. The immune complexes were detected with horseradish peroxidase-conjugated secondary antibodies, using enhanced chemiluminescence (Amersham Pharmacia, Piscataway, NJ, USA) with exposure to light-sensitive film (Amersham Pharmacia, Piscataway, NJ, USA). The band intensity was determined by a computer image analysis system (AI680, GE Healthcare, Uppsala, Sweden).

### 4.9. RNA Extraction and Real-Time RT-PCR

Total RNA was extracted from kidney tissue and cells using TRIzol (Invitrogen, Waltham, MA, USA). cDNA was synthesized using oligo d(T)16 and the SuperScript III Reverse Transcriptase kit (Invitrogen). Real-time PCRs were performed with SYBR green premix buffer and a LightCycler 480II (Roche, Basel, Switzerland). The housekeeping gene Cycophyilin A (CypA) was used as a control to normalize the mRNA content of each sample, as described in the manual. Relative expression levels were determined after normalization of the threshold cycle (Cr) values for CypA.

### 4.10. Statistical Analysis

All data are presented as the mean ± SD. Statistical significance was determined using one-way analysis of variance (ANOVA) followed by a post hoc Duncan’s method for multiple comparisons. Differences in *p*-values < 0.05 or 0.01 were considered statistically significant. The results were analyzed using SPSS 24.0 program (SPSS, Inc., Chicago, IL, USA).

## 5. Conclusions

The results obtained with the hydro-ethanoic dry extract of *R. sachalinensis* roots showed an important potential to be explored for BPH treatment. Thus, from what is known so far, the results of this study showed for the first time a stabilized product, which provides consistent information showing lower prostate weight and attenuation of the histopathological alterations induced by testosterone in the HKC-treated group. Therefore, the data suggested that the administration of an HKC could reduce the prostate in DHT in serum and prostate. As well, the HKC-treated group showed a significant reduction of 5-AR gene expression, and testosterone tended to increase. That implies the restoration of the normal prostate tissue. In conclusion, these findings elucidate that HKC extract under investigation has good potential for drug discovery in BPH future treatments.
